# Clinical Clues to Avoid Missing Melanoma When Morphology is Not Enough

**DOI:** 10.5826/dpc.1104143

**Published:** 2021-10-01

**Authors:** Giuseppe Argenziano, Giulia Briatico, Gabriella Brancaccio, Roberto Alfano, Elvira Moscarella, Aimilios Lallas

**Affiliations:** 1 Dermatology Unit, University of Campania Luigi Vanvitelli, Naples; 2 Department of Anesthesiology, Surgery and Emergency, University of Campania Luigi Vanvitelli, Naples; 3 First Dermatology Department, Medical School, Aristotle University of Thessaloniki, Thessaloniki, Greece

**Keywords:** melanoma, dermoscopy, diagnosis

This editorial on difficult-to-diagnose melanomas will discuss the following points:

1. Melanoma can be missed not only when the lesion is lacking specific morphologic clues, but also when the lesion is localized on covered areas and the patient is not undressed by the clinician.
2. When morphologic criteria to diagnose melanoma are lacking, there are 5 particularly relevant clinical clues to be considered, in order to avoid the risk of leaving a melanoma untreated.
3. Once the lesion is excised, melanoma could still be missed if a careful clinico-pathologic correlation is not carried out.

## No One Should Die of Melanoma

“No one should die of melanoma”. The reason why AB Ackerman wrote such a catchy statement back in 1985 [1] is related to the fact that, at least theoretically, all melanomas can be recognized and treated at an early stage because of their location on the skin, which is easy to be examined. Unfortunately, many people still die of melanoma, and this is due to at least 3 main actors.

## The First Actor is Melanoma Itself

There is a small number of tumors that are typified by a highly aggressive behavior. This type of melanomas, namely nodular melanomas, develop fast and become thick enough to acquire the potential to metastasize in few months only [2,3]. Nodular melanomas are difficult to excise before they become dangerous, thus very little could be done to change this dramatic scenario.

## The Second Actor is the Patient

In contrast to the previous and, fortunately, more rare melanoma type, the largest proportion of melanomas are slow growing and need years to acquire the potential to metastasize [4]. Thus, the largest amount of melanomas could be treated at early stages if patients were able to realize that they need medical advice. Unfortunately, many patients do not know that something wrong is going on because melanomas grow silently and do not give symptoms before it is too late. This scenario could potentially be changed if specific campaigns were in place to encourage anyone noticing a changing skin lesion to seek for medical advice.

## The Third Actor is the Doctor

A melanoma might be fatal when it grows too fast, when the patient waits too long before seeking consultation, but also when it is overlooked by the clinician. Clinicians could easily and efficiently improve this scenario by thoroughly distinguishing potentially dangerous cases, aiming to rule out any missed melanoma diagnosis. A first potentially dangerous case occurs when the patient is not undressed and melanoma is located on his/her covered body areas, therefore limiting a thorough investigation. The second potentially dangerous case occurs when a melanoma is left untreated because it morphologically mimics a benign lesion. Finally, the third dangerous case might take place when melanoma is biopsied by the clinician, but the correct diagnosis is not made histopathologically.

## Undress High Risk Patients and Dermoscopically Examine All Lesions

Nowadays, only a minority of physicians perform total body skin examination (TBSE) of all patients [5,6]. This is due to time constraint, cultural attitudes, and perhaps an insufficient knowledge of the potential threat caused by this behavior. A few years ago we performed a study showing that to detect 1 skin malignancy 47 patients need to be examined by TBSE, and 400 patients need to be examined to detect 1 melanoma [7]. In other words, a physician visiting 20 patients per day might miss a melanoma every 20 working days if he/she does not perform TBSE on all patients. Factors significantly increasing the chance to detect a skin cancer included age, male gender, a previous non-melanoma skin cancer, fair skin type, a skin tumor as the reason for consultation, and the presence of an equivocal lesion on uncovered areas.

Therefore, it is of great importance to continuously highlight the fact that a significant number of melanomas could be diagnosed earlier if TBSE is performed and all lesions are examined using our dermatoscope. This second suggestion derives from many studies [8,9], and long personal experience demonstrating that a significant number of melanomas appear clinically as benign and only dermoscopy may increase our index of suspicion. This is one of the main values of dermoscopy, namely, allowing the visualization of melanoma features also in very early melanomas that are still too small to have had time to develop the clinical criteria for a correct diagnosis [10].

## Use 5 Clinical Clues Coupled with Dermoscopy

Unfortunately, there is a number of melanomas that are morphologically inconspicuous, not only clinically, but also dermoscopically [11,12]. This is the worst scenario that all physicians, who are routinely involved in skin cancer screening, have experienced several times. The scenario might be eventually considered less severe when dealing with a difficult-to-diagnose melanoma in situ, but it turns dramatic in the context of a thick melanoma that is morphologically banal. When there is a lack of morphologic criteria to diagnose melanoma, there are 5 particularly relevant clinical clues to reduce the risk of leaving a melanoma untreated.

### 1. Patient Age

**Figure 1:**
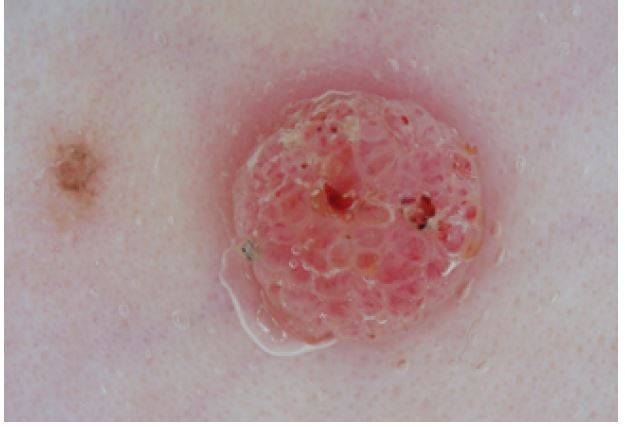
Dermoscopic view of a pink lesion showing papillomatous structures and polymorphic vessels.

**Figure 2:**
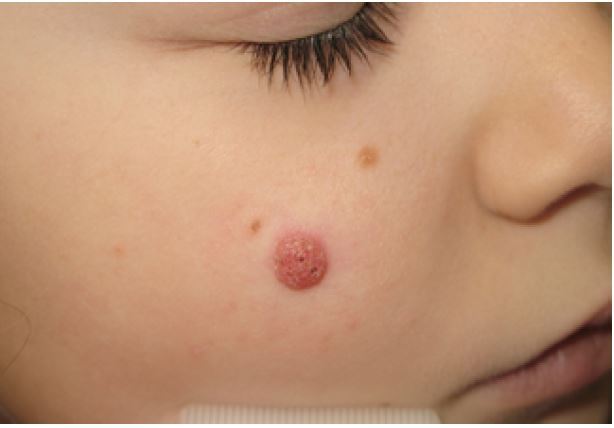
Clinical view of the lesion depicted in figure 1.

**Figure 3:**
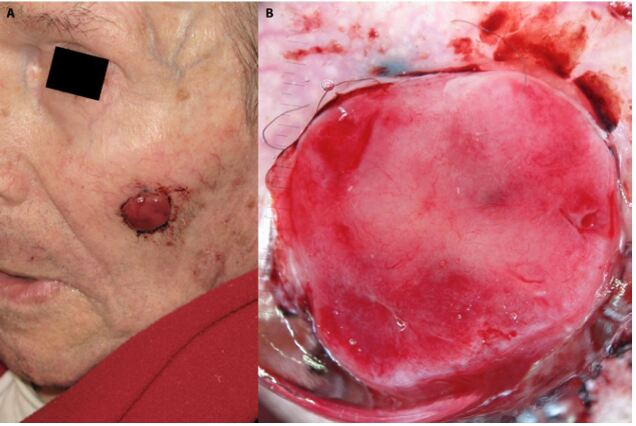
Clinical and dermoscopic view of an ulcerated nodular melanoma.

Patient age is, in our estimation, the strongest single clinical information influencing the clinician’s decision making. A good example is given in Figure 1, in which a non-pigmented lesion is depicted. Dermoscopically, there is a papillomatous pattern of irregular vessels pointing toward several differential diagnoses, including dermal nevus and seborrheic keratosis, but even melanoma cannot be excluded. However, in Figure 2 the clinical picture reveals a lesion located on the face of a prepubertal child. This, in addition to the dermoscopic morphology, allows to accurately diagnose a Spitz nevus. In Figure 3, the clinical and dermoscopic scenario of an ulcerated, nodular lesion located on the face of an elderly man leads immediately to the exclusion of a Spitz nevus case. In this context, one must decide what kind of malignant tumor this might be and, based on the additional clue of thin polymorphic vessels and a spot of bluish color at the periphery, melanoma diagnosis could be suggested. Thus, when a lesion is difficult to diagnose morphologically, the patient age is the first clinical clue to be considered.

### 2. Patient Sex

**Figure 4:**
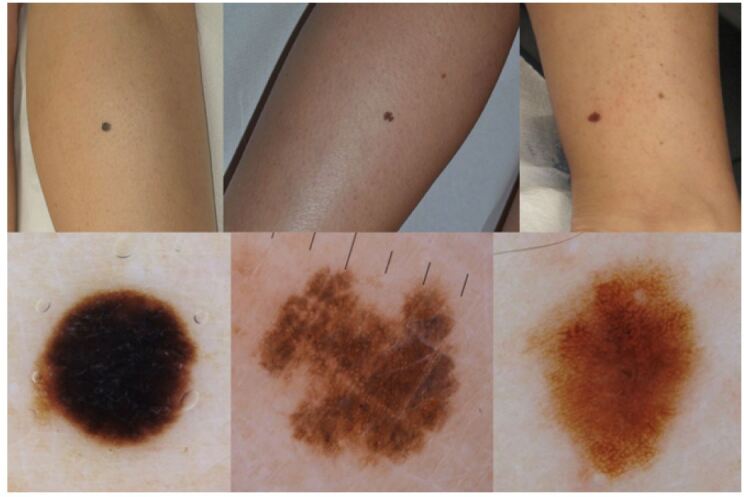
Clinical and dermoscopic views of 3 melanomas in situ located on the leg of 3 middle-aged women. Dermoscopic examination reveals 3 relatively symmetric lesions with only slightly atypical features. The lesions were finally excised because they were clinically solitary and located on the leg of middle-aged women.

Patient gender is particularly relevant in the context of inconspicuous melanocytic lesions located on middle-aged women’s legs. It is commonly known that the most frequent location of melanoma in females is the lower limbs [13], thus our index of suspicion should increase in these cases, especially in the context of solitary melanocytic proliferations of the legs of middle aged or elderly women (Figure 4).

### 3. Lesion Location

**Figure 5:**
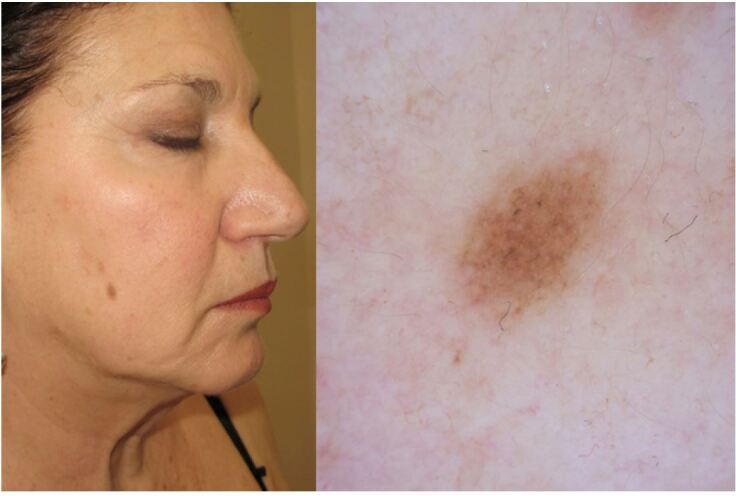
Clinical and dermoscopic view of a melanoma in situ, seen on the cheek of a 50-year-old woman who came for the treatment of the lesion due to cosmetic reasons. Dermoscopically, the lesion is suspicious because of the absence of benign features.

The specific anatomic location of the lesion is another striking clinical clue influencing final clinical judgment. There are skin body areas in which differential diagnosis is more difficult than others from a morphologic point of view. Flat facial lesions, for instance, are extremely difficult to differentiate due to a number of factors, including the different anatomy of the skin (the flat dermo-epidermal junction of the facial skin vs. the undulated junction of the trunk and limbs), the absence of melanoma-specific features in virtually all very early facial in situ melanomas, and the fact that patients with early facial melanoma frequently seek consultation for cosmetic reasons and not for the fear of a dangerous lesion (Figure 5) [14]. To minimize the risk of missing a melanoma, a specific rule should be applied for the evaluation of flat facial lesions. This is called “the inverse approach” and consists of searching, first, for 6 specific benign features [15, 16]. If none of the 6 benign features are clearly seen covering most of the lesion surface, the given lesion must be considered suspicious independently from the presence of melanoma-specific features. Using this diagnostic approach allows 84% sensitivity for melanoma with 86% specificity, representing, for the time being, the best approach to manage flat lesions on this difficult body area.

**Figure 6:**
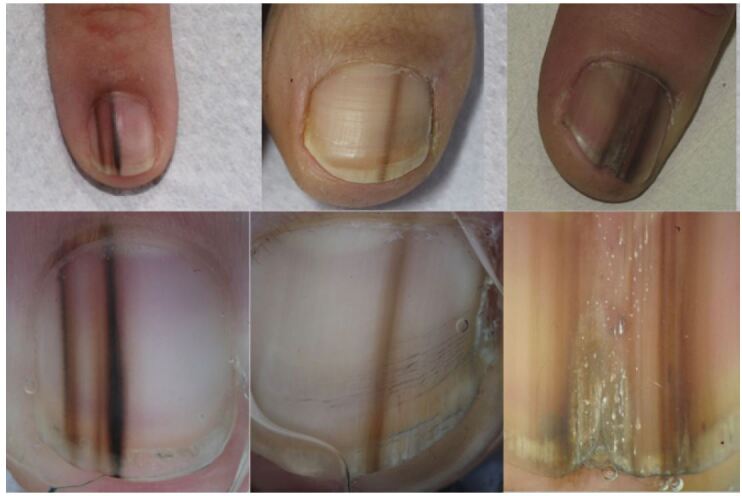
Three clinico-dermoscopic scenarios of pigmented nail bands. On the left a 9-year-old child with an irregularly pigmented, congenital melanocytic nevus. In the center a 37-year-old man with a thin and regular nail band favoring the diagnosis of melanocytic nevus. On the right a 43-year-old man with a melanoma in situ seen as a large and irregularly pigmented longitudinal melanonychia.

Another special location worth to mention is the nail. Nail melanomas involve nail matrix melanocytes in most cases, thus by definition they morphologically appear as pigmented nail bands. In the context of the so called longitudinal melanonychia, the differential diagnosis might be extremely difficult, calling again for specific rules. Here, only 3 clinico-dermoscopic scenarios should be considered. First, a child with a pigmented nail band. In this scenario, the most probable diagnosis is congenital melanocytic nevus of the nail matrix, and this diagnosis can be made with confidence also in the case of a large and irregular nail band. The second scenario involves an adult patient with a small longitudinal melanonychia. In this case the most probable diagnosis is nevus but 2–3 years follow-up should be carried out to rule out a very early melanoma. The third scenario is that of an adult patient with a large (more than 1/3 of the nail plate) pigmented nail band (Figure 6). In this case, melanoma is the most probable diagnosis, thus the lesion should be immediately biopsied, independently from the presence of regular or irregular coloration of the band [17, 18].

The last special location where, as a rule, we need to combine clinical and dermoscopic criteria, is the mucosal area. The most frequent benign pigmented lesion in this location is mucosal melanosis that is usually observed as a clinically flat lesion with dermoscopic parallel lines [19]. Conversely, melanoma is most frequently appearing as a palpable lesion, dermoscopically typified by a structureless pigmentation varying from blue to white and red [20].

### 4. Patient’s Lesions Comparison

**Figure 7:**
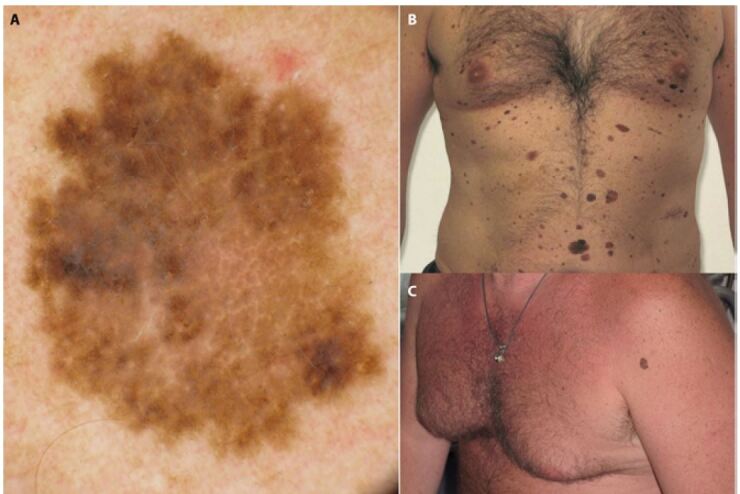
(A) Dermoscopic view of an irregularly pigmented lesion that could be judged as a lesion to be monitored if belonging to a patient with multiple nevi. (B) The lesion belongs to a patient with only few nevi, (C) therefore it was accordingly judged as to be excised. Subsequent histopathologic examination revealed a melanoma in situ.

The 3 rules to be used not to miss a melanoma in patients with multiple nevi are the following: (i) examine all lesions, (ii) use the comparative approach, and (iii) monitor the patient over time [21, 22]. The comparative approach is an extremely important strategy to minimize unnecessary excisions while selecting the real suspicious ones [21]. In general, different benign lesions within the same patient, appear very similar, whereas melanoma is frequently morphologically different from the rest of the nevi of the same patient. In addition, in patients with multiple atypical nevi, the threshold for excision should be set to a higher level compared to the lower threshold used in a patient with a solitary atypical lesion, as depicted in Figure 7.

### 5. Palpable and/or Pink Lesions

**Figure 8:**
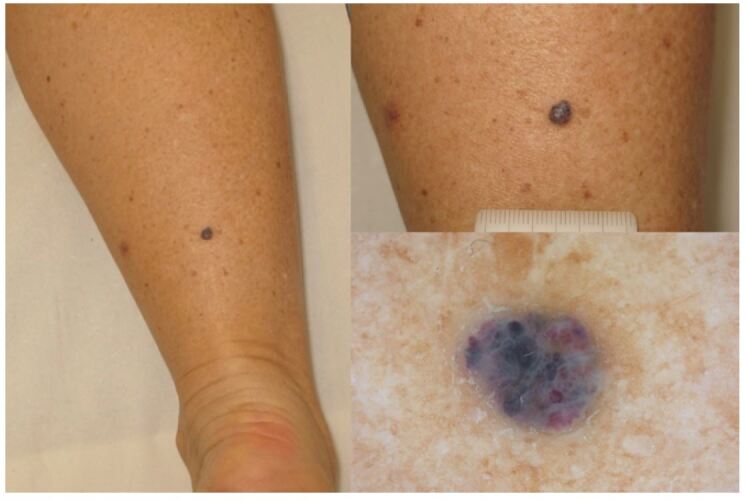
Clinical and dermoscopic view of a palpable lesion with stereotypical blue-red lacunas as seen in a hemangioma. The diagnosis of a benign lesion can be made with confidence and no further action is needed.

**Figure 9:**
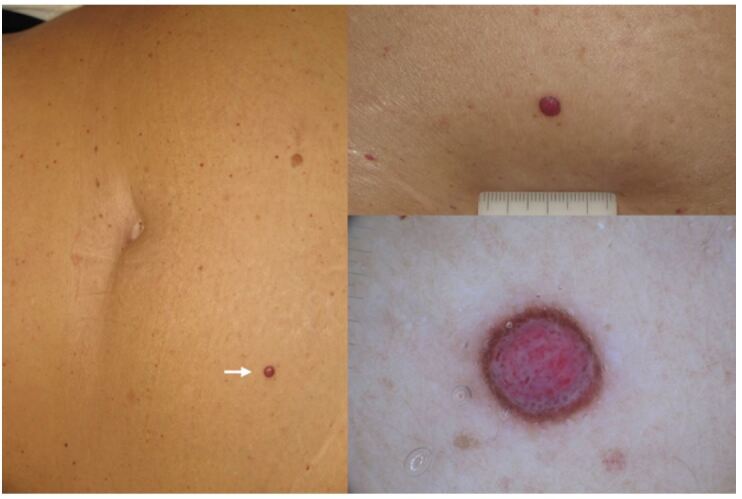
Clinical and dermoscopic view of a palpable lesion with symmetric distribution of colors and structures. Diagnosis of a benign lesion could not be made with confidence (several diagnostic options could be considered), therefore the lesion had to be excised with no monitoring. Subsequent histopathologic examination revealed a melanoma of 3.5 mm of thickness.

Patients with multiple nevi are the main indication for a digital monitoring procedure. A solitary doubtful lesion should be usually excised but, in the context of a macular lesion, a short-term digital follow-up is a valid alternative [22, 23]. In contrast, a palpable lesion should be excised immediately when the diagnosis of a benign lesion is not straightforward. This rule should always be applied not to miss a potentially aggressive melanoma (Figures 8 and 9).

The same behavior should be applied for pink tumors. The latter is still a very difficult diagnostic area in the realm of skin cancer screening. Although for basal cell carcinoma and, more recently, for squamous cell carcinoma, specific and reliable diagnostic criteria have been described [24], in the context of amelanotic melanomas there are only few and subtle diagnostic clues to look for. Since amelanotic melanoma might be potentially fatal, any pink tumor that cannot be clearly diagnosed as a benign lesion should be promptly excised.

#### Why is Clinical History not Relevant?

**Figure 10:**
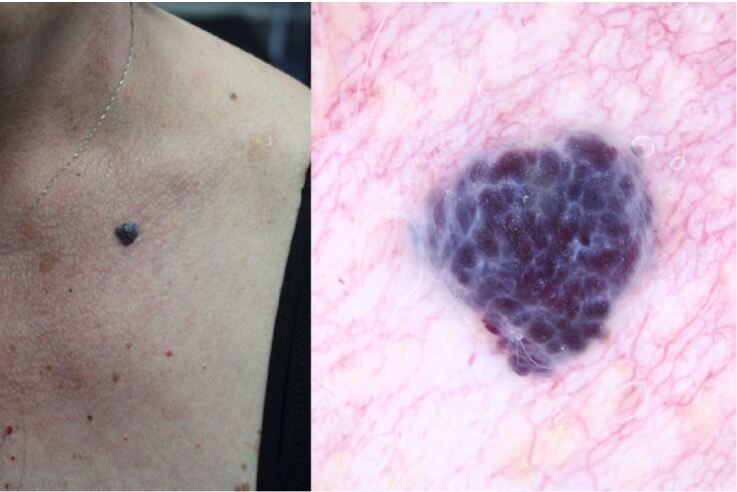
Clinical and dermoscopic view of a palpable lesion with stereotypical blue-red lacunas as seen in a hemangioma. The patient was concerned by the lesion’s recent onset and by its rapid changes. In this case the history was a confounding factor.

**Figure 11:**
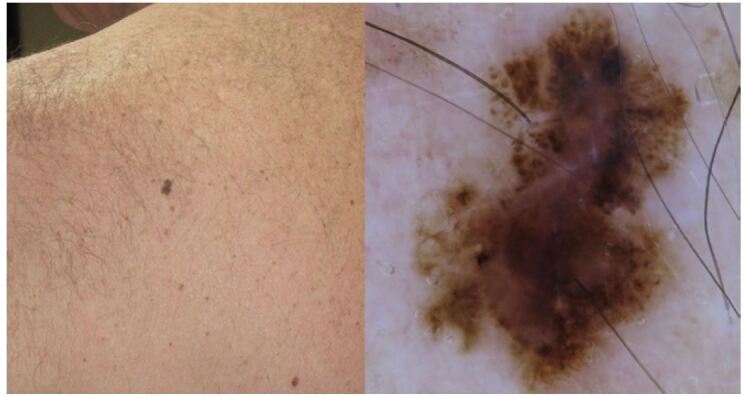
Clinical and dermoscopic view of a small but largely atypical melanocytic lesion morphologically suggestive of melanoma. The patient’s wife reported the lesion as a long-standing stable macule. Again, the history was a confounding factor because the lesion was excised and diagnosed as an early invasive melanoma histopathologically.

**Figure 12:**
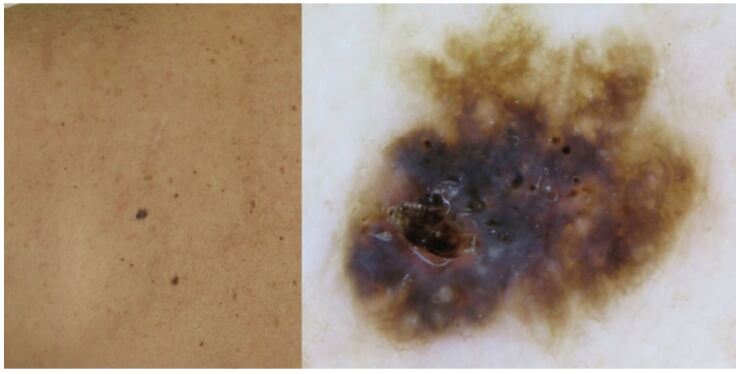
Clinical and dermoscopic view of a pigmented lesion showing some degree of conflicting features. A differential diagnosis between seborrheic keratosis and melanoma should be considered. The final management decision depends on the “objective” diagnostic confidence of the clinician and not on a “subjective” variable as the clinical history. The lesion was finally excised with subsequent histopathologic examination revealing a seborrheic keratosis.

Among the five clinical information that can be relevant to diagnose a morphologically inconspicuous melanoma, the clinical history is not mentioned on purpose. It is generally recognized that taking a good history is of uppermost importance for a reliable differential diagnosis but, in our estimation, this is not a valid concept in the context of skin cancer screening. As shown in Figures 10 and 11, clinical history might be a potentially confounding feature when a confident diagnosis can be rendered based on objective morphologic criteria. Conversely, when the given lesion is inconspicuous from a morphological point of view, the final decision cannot be based on a subjective criterion as clinical history (Figure 12). Patient’s clinical history certainly remains a mainstay, but not the history related to the single examined lesion.

#### Combine Clinical & Histopathologic Information

**Figure 13:**
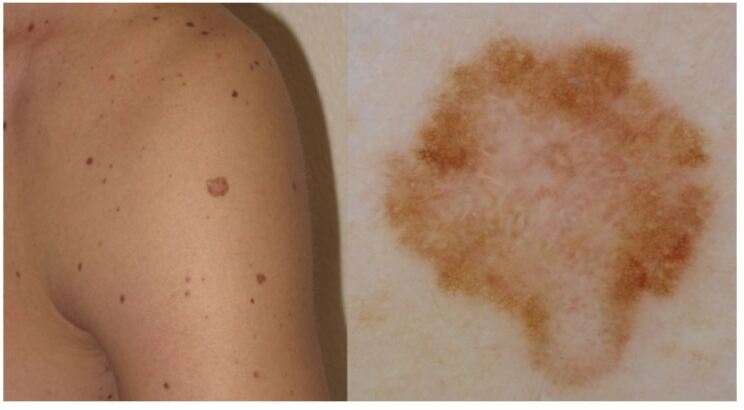
Clinical and dermoscopic view of an irregularly pigmented melanocytic lesion in a 37-year-old man.

**Figure 14:**
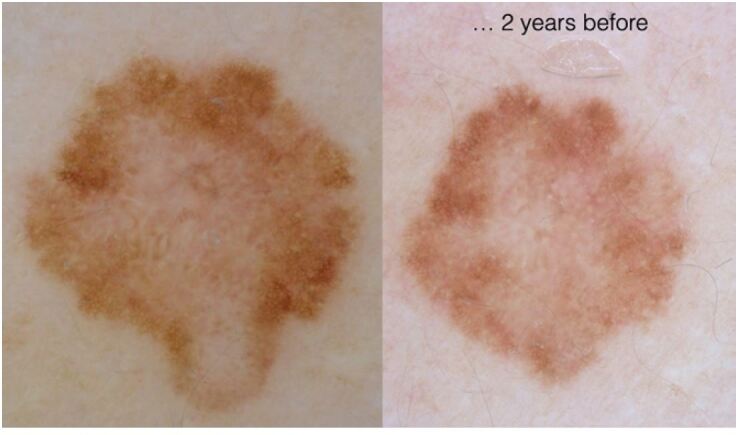
Side-by-side image comparison of the lesion depicted in figure 13.

**Figure 15:**
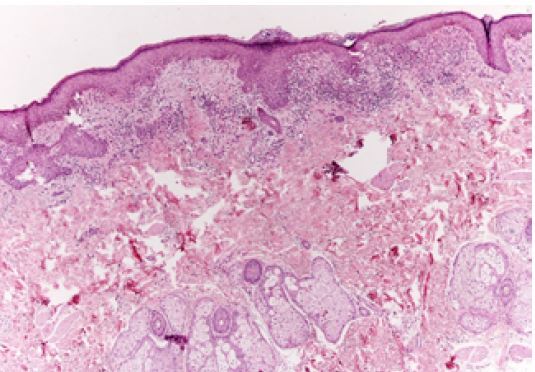
Histopathologic view of the lesion shown in figure 13.

The last step in the management of a given lesion, in which a melanoma could be overlooked, relies on histopathologic diagnosis. In Figure 13 a clinical and dermoscopic atypical lesion is depicted. As seen by the side-by-side image comparison (Figure 14), the lesion changed strikingly over a 24-month monitoring period. The lesion was excised, and a subsequent histopathologic diagnosis of dysplastic nevus was rendered (Figure 15). However, based on a clinico-pathologic discussion, in which the pathologist was made aware of the striking changes of the lesion over time, the diagnosis changed to melanoma in situ. Since a clinico-pathologic discussion is possible only if images of the given lesion are available, the rule is to take a good clinico-dermoscopic documentation of all lesions undergoing excision and subsequent histopathologic examination.
